# Integrating Radiomics and Lesion Mapping for Cerebellar Mutism Syndrome Prediction

**DOI:** 10.3390/children12060667

**Published:** 2025-05-23

**Authors:** Xinyi Chai, Wei Yang, Yingjie Cai, Xiaojiao Peng, Xuemeng Qiu, Miao Ling, Ping Yang, Jiashu Chen, Hong Zhang, Wenping Ma, Xin Ni, Ming Ge

**Affiliations:** 1 Department of Neurosurgery, Beijing Children’s Hospital, Capital Medical University, National Center for Children’s Health, Beijing 100045, China; chaixinyi1999@163.com (X.C.); dr.yangwei@foxmail.com (W.Y.);; 2 Department of Urology, Beijing Chao-Yang Hospital, Capital Medical University, Beijing 100024, China; 3 Institute of Urology, Capital Medical University, Beijing 300211, China; 4 Department of Image Center, Beijing Children’s Hospital, Capital Medical University, National Center for Children’s Health, Beijing 100045, China; 5 Department of Otolaryngology Head and Neck Surgery, Beijing Children’s Hospital, Capital Medical University, National Center for Children’s Health, Beijing 100045, China; nixin@bch.com.cn

**Keywords:** cerebellar mutism syndrome, lesion–symptom mapping, radiomics, predictive model, machine learning

## Abstract

**Objective:** To develop and validate a composite model that combines lesion–symptom mapping (LSM), radiomic information, and clinical factors for predicting cerebellar mutism syndrome in pediatric patients suffering from posterior fossa tumors. **Methods:** A retrospective analysis was conducted on a cohort of 247 (training set, *n* = 174; validation set, *n* = 73) pediatric patients diagnosed with posterior fossa tumors who underwent surgery at Beijing Children’s Hospital. Presurgical MRIs were used to extract the radiomics features and voxel distribution features. Clinical factors were derived from the medical records. Group comparison was used to identify the clinical risk factors of CMS. Combining location weight, radiomic features from tumor area and the significant intersection area, and clinical variables, hybrid models were developed and validated using multiple machine learning models. **Results:** The mean age of the cohort was 4.88 [2.89, 7.78] years, with 143 males and 104 females. Among them, 73 (29.6%) patients developed CMS. Gender, location, weight, and five radiomic features (three in the tumor mask area and two in the intersection area) were selected to build the model. The four models, KNN model, GBM model, RF model, and LR model, achieved high predictive performance, with AUCs of 0.84, 0.83, 0.81, and 0.87, respectively. **Conclusions:** CMS can be predicted using MRI features and clinical factors. The combination of radiomics and tumoral location weight could improve the prediction of CMS.

## 1. Introduction

Posterior fossa tumors represent the most common type of central nervous system (CNS) tumors in children [1]. Approximately 25–40% of children with posterior fossa tumors develop cerebellar mutism syndrome (CMS) [2]. CMS is characterized by significant linguistic impairment, often accompanied by cerebellar motor syndrome and cerebellar cognitive affective syndrome [3,4]. While mutism is typically transient and usually recoverable within 1–3 months for most patients, long-term side effects can negatively affect the quality of life for patients for a long period of time [5]. Early diagnosis and intervention can facilitate early rehabilitation.

The precise mechanism of CMS remains unclear. Midline location and medulloblastoma [6,7] have been identified as risk factors for CMS in numerous studies, while the significance of gender and tumor size remains unclear [8]. Lesion–symptom mapping (LSM) is a technique used to link lesion areas to clinical outcomes and generate lesion maps accordingly. Previously, we investigated the relationship between postoperative CMS and the tumor location and generated a lesion map via LSM [9]. We subsequently transformed the lesion map into a coefficient matrix to quantify the contribution of each voxel to CMS prediction. Integrating the tumor location weight with significant clinical variables, we developed a predictive model for CMS [10], which demonstrated satisfactory performance in predicting CMS. However, the intrinsic characteristics of the tumor were not incorporated thoroughly.

Radiomics converts the medical images into high-throughput quantitative features, enabling detailed characterization of the three-dimensional (3D) morphology and grayscale distribution in the region of interest [11,12]. This approach has shown great potential in characterizing tumor phenotypes, predicting diagnosis and outcomes [13]. By integrating radiomic features, location weights, and baseline clinical features, we aim to enhance the accuracy of CMS prediction for pediatric posterior fossa tumor patients. As a result, the aim of this study is to develop a unified prediction model incorporating the three aspects mentioned above to improve performance and offer a more resilient tool for CMS prediction.

## 2. Materials and Methods

### 2.1. Study Design and Participants

This is a retrospective cohort study. The study includes patients who were diagnosed with a posterior tumor and underwent surgical treatment at Beijing Children’s Hospital from June 2013 to December 2023. The patients were randomly divided 7:3 into a training set of 174 and a validation set of 73.

Inclusion criteria were as follows:

(1) Patients aged between 0 and 18 years;
(2) Completion of presurgical MRI at our center;
(3) Diagnosis of posterior fossa tumor confirmed at our center;
(4) Received surgical resection for the tumor;
(5) Definitive diagnosis of CMS or non-CMS determined by two senior neurosurgeons.

Exclusion criteria were as follows:

(1) Incomplete clinical data;
(2) Missing MRI data;
(3) Unsatisfactory normalization upon visual inspection.

The study was approved by our institution’s Ethics Committee. The requirement for individual informed consent was waived due to the retrospective nature of this study.

### 2.2. Imaging Procedures, Lesion Map Extraction, and Voxel Values Calculation

All images were acquired prior to the surgery. All patients from our cohort were scanned on a 3.0 T Discovery 750 scanner (GE Healthcare, Milwaukee, WI, USA) with an 8-channel head coil (voxel size, 0.6 × 0.6 × 5 mm^3^) or a 3.0 T Ingenia CX scanner (Philips Healthcare, Best, The Netherlands) with a 32-channel head coil (voxel size, 1 × 1 × 1 mm^3^). Our analysis of radiomic features from 15 patients scanned at both 1 mm and 5 mm slice thicknesses showed no significant thickness effect (*p* = 0.68), supporting inclusion of both thicknesses.

Tumor masks, excluding the peritumoral edema area, were delineated by a senior neurosurgeon on plain T1W images using ITK-SNAP software (version 4.0.1). These masks were subsequently reviewed by a senior radiologist in a double-blind manner. Both the neurosurgeon and the reviewing radiologist had no involvement in surgical procedures or clinical follow-up of the study cohort. Any discrepancies were resolved through discussion and consultation, with 12 cases requiring discussion for final boundary determination. T2W and FLAIR images were referred to during the delineation process. The scan images and tumor masks were then normalized into the Montreal Neurological Institute (MNI) space using the Clinical Toolbox in statistical parametric mapping (SPM12, Welcome Department of Neuroscience, London, UK. The normalized images were visually inspected, and the ones not well merged were excluded from further analysis.

A lesion map was created using the sparse canonical correlation analysis (SCCAN) method implemented in R Lesion-to-Symptom Mapping (LESYMAP). This method employs a multivariate strategy to perform LSM analysis. Specifically, the normalized tumor masks and CMS status were input into LESYMAP to generate a lesion map reflecting the voxel distribution associated with CMS and the strength of the correlation of each voxel with CMS. In this study, we incorporated the lesion map generated in our previous study [9].

Subsequently, the lesion map built in our previous study was converted to the coefficient matrix S, which represented the voxel’s distribution and weight. The tumor mask for each patient was converted to a matrix denoted by M by Matlab. Weighted summation based on these two matrices was calculated to represent the effect of tumor location on each patient’s CMS status. The formula for the location weight is given by:

Location Weight = ∑∑∑(M × S)

### 2.3. Radiomic Feature Extraction and Selection

Feature extraction was performed using the open-source pyradiomics package in the 3D Slicer extension manager [12]. Herein, we defined the intersection area as the overlap between the significant region map and the individual tumor mask for each patient in MNI space. The significant region map represents areas significantly associated with CMS calculated by SCCAN. A total of 1573 features were extracted from T1W in the tumor mask area and the intersection area, respectively. The extracted features were standardized using the Min-Max normalizer to ensure comparability and to mitigate the impact of scale differences.

The following two steps were used to select the best radiomic features. First, the radiomic features were screened by Student’s *t*-test, with a significance of *p* < 0.05. Subsequently, the least absolute shrinkage and selection operator (LASSO) regression algorithm and penalty parameter adjustment were used for 10-fold cross-validation. The optimal feature data set with the smallest cross-validation binomial deviation was selected.

### 2.4. CMS Definition and Risk Factors

The clinical records were meticulously reviewed. CMS was defined as mutism or severe reduction of speech after surgery. Additional symptoms might include hypotonia, dysphagia, irritability, and involuntary movement. All patients received at least one follow-up, during which the duration of mutism was recorded to aid the diagnosis of CMS. The clinical variables included in this study were age at surgery, tumor size, tumor consistency, hydrocephalus, paraventricular edema, presurgical VP shunt, surgical route, gender, pathology, and midline location. Tumor size was defined as the maximum diameter of three planes. A tumor with a solid component comprising more than half of its total volume was defined as a solid tumor. Surgical routes were classified into telovelar approach, trans-vermis approach, other surgical approach, and unknown approach. Paraventricular edema was reviewed on the FLAIR image. The pathologies were separated into two categories: non-medulloblastoma (non-MB) and medulloblastoma (MB).

Continuous and categorical variables were compared between CMS and non-CMS groups using the Kruskal–Wallis and chi-square tests, respectively. *p* < 0.05 was the threshold for statistical significance. Significant variables were included in the predictive model. Confounding variables were excluded.

### 2.5. Model Development and Validation

We examined clinical variables and radiomic features and calculated location weight before performing cross-validation to identify machine learning models with the best performance. We fitted the following models: KNN, GBM, RF, and LR on the training set. The validation cohort was used to validate the model’s prediction performance. The area under the curve (AUC) of the Receiver Operating Characteristic (ROC) curves was calculated to analyze the model’s performance. The Hosmer–Lemeshow goodness-of-fit test was utilized to assess the calibration of the prediction model.

### 2.6. Statistical Analysis

Statistical analysis was performed by Matlab (R2017b), R4.3.3 (https://www.R-project.org) and Python 3.7. Continuous variables were described as medians and quartiles, while categorical variables were described as frequencies.

## 3. Results

### 3.1. Baseline Characteristics of the Study Cohorts

A total of 247 patients (mean age at surgery, 4.88 [2.89, 7.78] years) were included, with 104 females and 143 males. Seventy-three (29.6%) were diagnosed with CMS. We randomly divided the patients into a training cohort of 174 (46 (26.4%) diagnosed with CMS) and a validation cohort of 73 (27 (37.0%) diagnosed with CMS) at a ratio of 7:3. The clinical data and location weight of the different cohorts are presented in Table 1. Baseline characteristics were well balanced between the training cohort and the validation cohort, with all variables showing no significant differences (*p*-value > 0.05).

### 3.2. Group Comparison of Non-CMS and CMS Cohorts

Between non-CMS and CMS cohorts, no significant difference was found in age at surgery (*p* = 0.146), tumor size (*p* = 0.825), tumor consistency (non-CMS: 83.9% vs. CMS: 90.4%, *p* = 0.255), hydrocephalus (non-CMS: 44.8% vs. CMS: 52.1%, *p* = 0.369), paraventricular edema (non-CMS: 58% vs. CMS: 69.9%, *p* = 0.11), and presurgical VP shunt (non-CMS: 9.2% vs. CMS: 12.3%, *p* = 0.607). However, significant differences were shown in gender (non-CMS: 53.4% vs. CMS: 68.5%, *p* = 0.041), surgical route (*p* = 0.01), pathology (non-CMS: 31.0% vs. CMS: 50.7%, *p* = 0.005), and midline location (non-CMS: 66.7% vs. CMS: 86.3%, *p* = 0.003) (Table 2). Among the significant variables, gender was selected as a presurgical risk factor in the prediction model. Midline location was excluded as this can be precisely reflected by the location weight. The surgical route was excluded from the model due to substantial missing information. MB was not included as the diagnosis could not be confirmed before surgery.

### 3.3. Feature Selection

**Figure 1:**
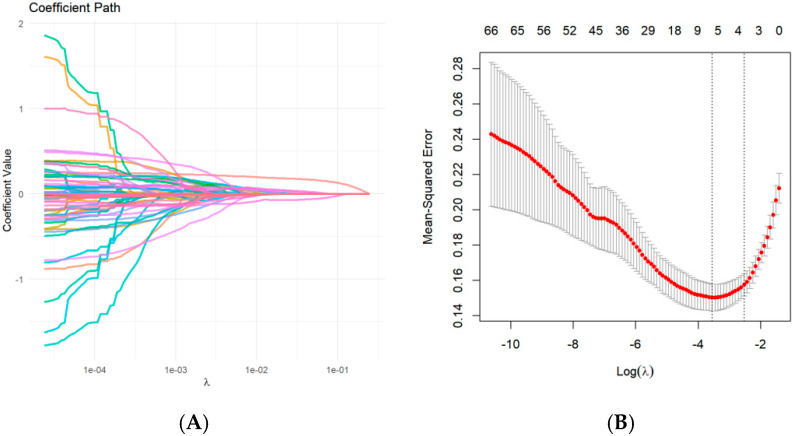
Screening of radiomic features based on Lasso regression: (**A**) the variation characteristics of the coefficient of variables; (**B**) the selection process of the optimum value of the parameter λ in the Lasso regression model by the cross-validation method.

A total of 3146 radiomic features were extracted from the T1W images in the tumor mask and the intersection area. In the first feature selection step, 224 features were roughly selected using an independent-samples *t*-test. Subsequently, Lasso regression determined 5 predictive radiomics features (“original_glcm_SumSquares” in the intersection area, “original_glszm_SizeZoneNonUniformityNormalized” in the intersection area, “original_glszm_LowGrayLevelZoneEmphasis”, “original_glszm_GrayLevelVariance”, and “original_glszm_SmallAreaEmphasis” in the tumor mask area) (Figure 1).

### 3.4. Model Construction and Performance

**Figure 2:**
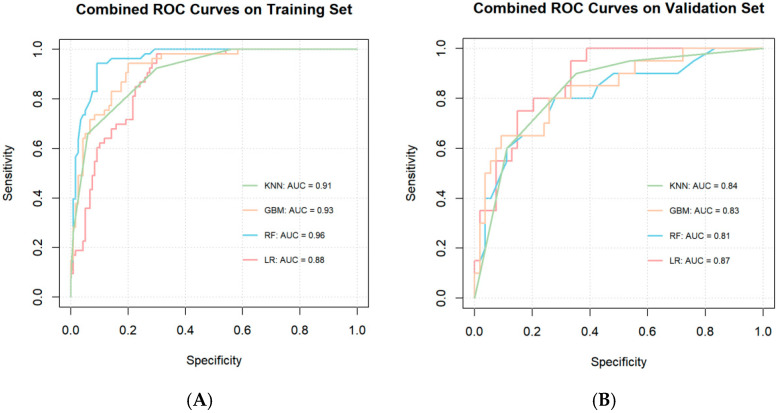
The ROC curves of the model on (**A**) the training set and (**B**) the validation set.

With the selected radiomic features, voxel values, and epidemiologic covariates, prediction models were fitted on four different machine learning models (KNN, GBM, RF, LR) with 10-fold cross-validation. All ML models yielded satisfying performance in both training set (AUC = 0.91, 0.93, 0.96, and 0.88 for KNN model, GBM model, RF model, and LR model, respectively) and validation set (AUC = 0.84, 0.83, 0.81, and 0.87 for KNN model, GBM model, RF model, and LR model, respectively) (Figure 2). According to the Hosmer–Lemeshow test, all four models had acceptable model fits with *p* > 0.05 (*p* = 0.32, 0.77, 0.52, and 0.91 for the KNN, GBM, RF, and LR models, respectively).

## 4. Discussion

CMS is a common challenge for pediatric patients with posterior fossa tumors due to its symptoms of debilitating social and communication deficits [14]. Early intervention and therapy have been reported to be effective for rehabilitation [15,16]. Therefore, CMS risk evaluation is crucial in clinical practice. In this study, we developed a CMS prediction model based on clinical variables, tumor location weight, radiomic features, and intersection radiomic features. The model achieved a satisfactory predictive performance.

Multiple models have been built in an attempt to predict CMS risk based on presurgical MRI and clinical variables. Liu et al. created a CMS rating system with an accuracy of 78% based on cerebellar hemisphere location of the tumor, cerebellar hemisphere invasion, bilateral middle cerebellar peduncle invasion, dentate nucleus invasion, and age at imaging [17]. Bae et al. improved the previous model to an accuracy of 87%, a sensitivity of 97%, and a specificity of 84% by including radiological diagnosis (pathologically), tumor location on MRI, brainstem invasion, middle cerebellar peduncle invasion, and superior cerebellar peduncle invasion as risk factors [18].

In the study of preoperative MRI images in CMS, the tumor location has been extensively researched due to its potential pathological mechanism. It is believed that CMS results from bilateral damage to the proximal efferent cerebellar pathways and the dentato-rubro-thalamo-cortical tracts [19,20,21]. The injury to these fibers is considered to interfere with remote supratentorial functions [22,23]. Temporary reduced metabolism in the vermis, thalami, caudate nuclei, and long-term prefrontal hypometabolism has been observed in CMS patients [24], indicating that the tumor location plays a critical role in CMS outcome. The voxels affecting CMS are sparsely distributed rather than clustered into a contiguous block; therefore, location information could be more precisely reflected at a voxel level [25]. In our previous study, we built a prediction model based on clinical variables and tumor location weight calculated by the weighted summation on each voxel in the tumor mask area based on the coefficient matrix developed in our study [9]. The matrix was derived by assessing the strength of correlation between voxels and CMS with LSM [10]. The model reached a satisfying performance; however, there is more information conveyed in the imaging data that remains unmined and underutilized.

Radiomic features are calculated by the same formula, and therefore, they have stable and repeatable features [11]. There is also potentially a wealth of information (shape, volume, intensity, etc.) able to be extracted from the MRI through the radiomics approach [26,27]. Wang et al. selected radiomic features to successfully classify pediatric posterior fossa tumor pathology types [28]. Studies have also shown radiomic-based information has the ability to convey information on tumor consistency, molecular classification, staging, and so on in brain tumors [29,30,31]. In this study, we included radiomic features as variables to enhance the accuracy and comprehensiveness of the model. In addition to extracting and selecting radiomic features in the region of interest (ROI) area, similar to the workflow in previous studies, we also conducted the same feature selection process on the intersection area. Our significant region map mainly contains areas located at the right lobule Crus I, lobules IV-V, and lobule VI. Previous studies have indicated that right cerebellar lobules VI-Crus I are associated with language processing [32]. Evidence has also suggested the link between right medial posterior cerebellum damage and transient language difficulties [33,34]. By including the intersection of the tumor mask area and a significant region area, we hope to stress the damage a tumor does to the previously identified language function-related area. Compared to tumor masks, the intersection area is more important to CMS prediction. Location weight only reflects the location information; however, the intersection radiomics can reflect many other non-locational information, such as texture, intensity, and so on.

“original_glcm_SumSquares” in the intersection area, “original_glszm_SizeZoneNonUniformityNormalized” in the intersection area, “original_glszm_LowGrayLevelZoneEmphasis”, “original_glszm_GrayLevelVariance”, and “ original_glszm_SmallAreaEmphasis” in the tumor mask area were selected as predictive factors. Among the 5 selected radiomics features, 4 were grey-level size zone (GLSZM) features, which are proper for regionally evaluating nonperiodic and heterogeneous textures [35]. original_glcm_SumSquares and original_glszm_SizeZoneNonUniformityNormalized were selected from the intersection area, and both were utilized to predict tumor outcomes in previous studies [36,37]. By including these radiomic features in the intersection area, the texture in the key CMS reducing region can be successfully quantified and stressed. original_glszm_LowGrayLevelZoneEmphasis and other mask area radiomic features have been indicated to represent texture in the tumor area and have been used to predict glioblastoma recurrence in previous studies [38,39]. Therefore, by including these features, the overall internal texture of the tumor can be comprehensively reflected, offering a more nuanced understanding of its biological characteristics and behavior.

During baseline analysis, gender, surgical route, pathology, and midline location were identified as significantly different between the CMS and non-CMS groups. We included gender and voxel values (representing location) in the final model. The surgical route was excluded due to the confounding effect of the tumor location and multiple missing data. Pathology types were excluded due to the presurgical nature of this model. Moreover, we believe the pathological information is included in the radiomic information [40].

However, there are several limitations in this study. First, this is merely a single-center study. The robustness of the model needs further verification due to a relatively small sample size. Moreover, radiomics is only extracted from the T1 MRI in this study, and other MRI images were not fully utilized.

## 5. Conclusions

In conclusion, this study enhances our understanding of CMS pathophysiology by integrating location, tissue properties (radiomics), and demographic characteristics in risk stratification. The proposed model provides a promising tool for presurgical risk assessment and enables more personalized interventions, potentially improving outcomes for children at risk for CMS. By highlighting the contributions of specific radiomic features, our work paves the way for future studies exploring the biological underpinnings of CMS-related regions.

## Figures and Tables

**Table 1:** A summary of the clinical features of the two sets.

		Overall (N = 247)	Training (N = 174)	Validation (N = 73)	*p*-Value
		247	174	73	
**CMS (%)**	Non-CMS	174 (70.4)	128 (73.6)	46 (63.0)	0.132
	CMS	73 (29.6)	46 (26.4)	27 (37.0)	
**Location weight [Q1, Q3]**		1.72 [0.14, 4.62]	1.72 [0.17, 4.14]	1.43 [0.14, 5.34]	0.593
**Age at surgery [Q1, Q3]**		4.88 [2.89, 7.78]	4.85 [2.61, 7.60]	5.06 [3.05, 8.37]	0.658
**Gender (%)**	Female	104 (42.1)	67 (38.5)	37 (50.7)	0.104
	Male	143 (57.9)	107 (61.5)	36 (49.3)	
**Size [Q1, Q3]**		48.25 [40.00, 55.53]	48.19 [38.50, 54.91]	48.72 [40.00, 57.80]	0.192
**Consistency (%)**	Non-solid	35 (14.2)	27 (15.5)	8 (11.0)	0.461
	Solid	212 (85.8)	147 (84.5)	65 (89.0)	
**Hydrocephalus, *n* (%)**	No	131 (53.0)	98 (56.3)	33 (45.2)	0.145
	Yes	116 (47.0)	76 (43.7)	40 (54.8)	
**Paraventricular edema (%)**	No	95 (38.5)	72 (41.4)	23 (31.5)	0.19
	Yes	152 (61.5)	102 (58.6)	50 (68.5)	
**Presurgical VP shunt (%)**	No	222 (89.9)	152 (87.4)	70 (95.9)	0.072
	Yes	25 (10.1)	22 (12.6)	3 (4.1)	
**Surgical Route (%)**	R1	63 (25.5)	48 (27.6)	15 (20.5)	0.411
	R2	58 (23.5)	43 (24.7)	15 (20.5)	
	R3	55 (22.3)	35 (20.1)	20 (27.4)	
	R4	71 (28.7)	48 (27.6)	23 (31.5)	
**Pathology (%)**	Other	156 (63.2)	114 (65.5)	42 (57.5)	0.297
	MB	91 (36.8)	60 (34.5)	31 (42.5)	
**Midline location (%)**	No	68 (27.5)	48 (27.6)	20 (27.4)	1
	Yes	179 (72.5)	126 (72.4)	53 (72.6)	

Abbreviations: CMS = cerebellar mutism syndrome, R1 = other surgical routes, R2 = telovelar approach, R3 = trans-vermis approach, R4 = unknown approach, MB = medulloblastoma. Note: Location weight, age at surgery, and tumor size are presented with median [Q1,Q3].

**Table 2:** Comparison of CMS and non-CMS groups.

		Overall(N = 247)	Non-CMS (N = 174)	CMS(N = 73)	*p*-Value
**Location weight [Q1, Q3]**		1.72 [0.14, 4.62]	0.85 [0.00, 3.36]	3.68 [1.73, 7.18]	<0.001
**Age at surgery (years), median [Q1, Q3]**		4.88 [2.89, 7.78]	4.59 [2.64, 7.62]	5.41 [3.67, 7.81]	0.146
**Gender, *n* (%)**	Female	104 (42.1)	81 (46.6)	23 (31.5)	**0.041**
	Male	143 (57.9)	93 (53.4)	50 (68.5)	
**Size [Q1, Q3]**		48.25 [40.00, 55.53]	47.94 [39.50, 55.70]	48.32 [40.00, 55.20]	0.825
**Consistency, *n* (%)**	Non-solid	35 (14.2)	28 (16.1)	7 (9.6)	0.255
	Solid	212 (85.8)	146 (83.9)	66 (90.4)	
**Hydrocephalus, *n* (%)**	No	131 (53.0)	96 (55.2)	35 (47.9)	0.369
	Yes	116 (47.0)	78 (44.8)	38 (52.1)	
**Paraventricular edema, *n* (%)**	No	95 (38.5)	73 (42.0)	22 (30.1)	0.11
	Yes	152 (61.5)	101 (58.0)	51 (69.9)	
**Presurgical VP shunt, *n* (%)**	No	222 (89.9)	158 (90.8)	64 (87.7)	0.607
	Yes	25 (10.1)	16 (9.2)	9 (12.3)	
**Surgical Route, *n* (%)**	R1	63 (25.5)	41 (23.6)	22 (30.1)	**0.001**
	R2	58 (23.5)	32 (18.4)	26 (35.6)	
	R3	55 (22.3)	48 (27.6)	7 (9.6)	
	R4	71 (28.7)	53 (30.5)	18 (24.7)	
**Pathology, *n* (%)**	Other	156 (63.2)	120 (69.0)	36 (49.3)	**0.005**
	MB	91 (36.8)	54 (31.0)	37 (50.7)	
**Midline location, *n* (%)**	No	68 (27.5)	58 (33.3)	10 (13.7)	**0.003**
	Yes	179 (72.5)	116 (66.7)	63 (86.3)	

Abbreviations: CMS = cerebellar mutism syndrome, R1 = other surgical routes, R2 = telovelar approach, R3 = trans-vermis approach, R4 = unknown approach, MB = medulloblastoma. Note: Location weight, age at surgery, and tumor size are presented with median [Q1,Q3].

## Data Availability

The data supporting this study are available upon request from the corresponding author due to privacy restrictions.
